# Supplementary material for: Treatment outcomes of Nigerian patients with tuberculosis: A retrospective 25-year review in a regional medical center
Source: PLoS One. 2020 Oct 29;15(10):e0239225. doi: 10.1371/journal.pone.0239225 (PMC7595370; doi:10.1371/journal.pone.0239225)
Supplement: S1 Appendix — (DOCX) [file pone.0239225.s001.docx]

**APPENDIX I** Hospital attendance and number of TB cases managed in a regional health centre

| SN | Year | Adult Attendance | Adult TB Cases | Children Visit | Children TB Cases | Total Hospital Attendance | Total TB Cases |
| --- | --- | --- | --- | --- | --- | --- | --- |
|  | 1992 | 35266 | 98 | 20464 | 9 | 55730 | 107 |
|  | 1993 | 32557 | 169 | 18276 | 34 | 50833 | 203 |
|  | 1994 | 21885 | 99 | 10576 | 7 | 32461 | 106 |
|  | 1995 | 21659 | 83 | 9123 | 7 | 30782 | 90 |
|  | 1996 | 27256 | 89 | 11796 | 17 | 39052 | 106 |
|  | 1997 | 28121 | 177 | 12150 | 23 | 40271 | 200 |
|  | 1998 | 25130 | 198 | 10581 | 19 | 35711 | 217 |
|  | 1999 | 26900 | 116 | 10290 | 16 | 37190 | 132 |
|  | 2000 | 29713 | 128 | 10684 | 12 | 40397 | 140 |
|  | 2001 | 32945 | 117 | 13150 | 17 | 46095 | 134 |
|  | 2002 | 28978 | 120 | 12853 | 15 | 41831 | 135 |
|  | 2003 | 29282 | 144 | 12382 | 16 | 41664 | 160 |
|  | 2004 | 30009 | 129 | 13035 | 9 | 43044 | 138 |
|  | 2005 | 33265 | 137 | 13770 | 10 | 47035 | 147 |
|  | 2006 | 37533 | 146 | 15530 | 18 | 53063 | 164 |
|  | 2007 | 41663 | 169 | 15488 | 12 | 57151 | 181 |
|  | 2008 | 40702 | 133 | 14805 | 15 | 55507 | 148 |
|  | 2009 | 37114 | 102 | 12746 | 11 | 49860 | 113 |
|  | 2010 | 31337 | 96 | 10944 | 10 | 42281 | 106 |
|  | 2011 | 28254 | 98 | 10426 | 4 | 38680 | 102 |
|  | 2012 | 21281 | 93 | 6650 | 6 | 27931 | 99 |
|  | 2013 | 26327 | 83 | 9625 | 7 | 35952 | 90 |
|  | 2014 | 31738 | 68 | 10407 | 5 | 42145 | 73 |
|  | 2015 | 35530 | 85 | 12510 | 10 | 48040 | 95 |
|  | 2016 | 39098 | 93 | 14374 | 11 | 53472 | 104 |
|  | 2017 | 44352 | 82 | 16030 | 12 | 60382 | 94 |
|  | Total | 817895 | 3052 | 328665 | 332 | 1146560 | 3384 |
|  | | | | | |  |  |
