# Supplementary material for: Treatment outcomes of Nigerian patients with tuberculosis: A retrospective 25-year review in a regional medical center
Source: PLoS One. 2020 Oct 29;15(10):e0239225. doi: 10.1371/journal.pone.0239225 (PMC7595370; doi:10.1371/journal.pone.0239225)
Supplement: S2 Appendix — (PDF) [file pone.0239225.s002.pdf]

Appendix 2. Factors associated outcomes: death outcome

Factors	Death		Unadjusted		Adjusted	
	Yes n(%)	No n (%)	OR (95% CI)	p-value	OR (95% CI)	p-value
Sex						
Male	185(9.7)	1717(90.3)	1.1 (0.85 – 1.3)	0.595	1.1 (0.83 – 1.3)	0.653
Female	136 (9.2)	1346(90.8)		Ref		
Age group						
Adult	303(9.9)	2749(90.1)	2.0 (1.2 – 3.1)	0.009*	1.9 (1.2 – 3.1)	0.011*
Children	18(5.4)	314(94.6)		Ref		
TB Classification						
PTB	275 (9.6)	2603(90.4)	1.1 (0.76 – 1.5)	0.742	1.1 (0.75 – 1.1)	0.779
EPTB	46 (9.1)	460 (90.9)		Ref		
Pre-treatment						
New	286 (9.4)	2771(90.6)	0.7 (0.26 – 2.1)	0.589	0.7 (0.25 – 2.0)	0.520
Relapse	8 (7.8)	94 (92.2)	0.6 (0.17 – 2.2)	0.456	0.6 (0.16 – 2.1)	0.408
Transfer IN	0 (0.0)	6 (100.0)	NA	1.0	NA	1.0
Default IN	4 (12.1)	29 (87.9)		Ref		
Failure IN	13 (9.8)	119 (90.2)	0.79 (0.24 – 2.6)	0.701	0.7 (0.22 – 2.4)	0.588
Unknown IN	10 (18.5)	44 (81.5)	1.6 (0.47 – 5.8)	0.434	1.5 (0.43 – 5.4)	0.511
HIV Status						

Positive	25 (16.4)	127 (83.6)	2.1 (1.3 – 3.4)	0.004*	2.1 (1.3 – 3.5)	0.003*
Negative	74 (8.7)	778 (91.3)	Ref			
Unknown	222 (9.3)	2158 (90.7)	1.1 (0.82 – 1.4)	0.577	1.1 (0.85 – 1.5)	0.405

******statistically significant at p < 0.05, ref : reference level, NA – not available.* Significant variables on bivariate analysis associated with death were gender, age, forms of TB, pre-treatment and HIV status were subjected to binary logistic regression with reference indicator as the female gender, children, extra-pulmonary tuberculosis, pre- treatment status of default and HIV negative respectively.
