# Supplementary material for: Treatment outcomes of Nigerian patients with tuberculosis: A retrospective 25-year review in a regional medical center
Source: PLoS One. 2020 Oct 29;15(10):e0239225. doi: 10.1371/journal.pone.0239225 (PMC7595370; doi:10.1371/journal.pone.0239225)
Supplement: S3 Appendix — (PDF) [file pone.0239225.s003.pdf]

Appendix 3. Factors associated with potentially unsatisfactory treatment outcome.

Factors	Potentially unsatisfactory treatment outcome		Unadjusted		Adjusted	
	Yes n(%)	No n (%)	OR (95% CI)	p-value	OR (95% CI)	p-value
		Sex				
Male	269 (14.1)	1633 (85.9)	1.4 (1.2 – 1.8)	0.001*	1.4 (1.1 – 1.7)	0.002*
Female	154 (10.4)	1328 (89.6)		ref		
		Age group				
Adult	388 (12.7)	2664 (87.3)	1.2 (0.85 – 1.8)	0.257	1.0 (0.69 – 1.5)	0.963
Children	35 (10.5)	297 (89.5)		ref		
		TB Classification				
PTB	383 (13.3)	2495 (86.7)	1.8 (1.3 – 2.5)	0.001*	1.7 (1.2 – 2.4)	0.004*
EPTB	40 (7.9)	466 (92.1)		ref		
		Pre-treatment				
Relapse	13 (12.7)	89 (87.3)	1.1 (0.62 – 2.0)	0.702	1.0 (0.56 – 1.8)	0.960
Transfer IN	33 (50.0)	33 (50.0)	7.7 (1.5 – 58.2)	0.013*	7.6 (1.5 – 38.4)	0.014*

Default IN	10(30.3)	23 (69.7)	3.3 (1.6 – 7.1)	0.002*	3.0 (1.4 – 6.5)	0.004*
Failure IN	36 (27.3)	96 (72.7)	2.9 (1.9 – 4.3)	< 0.001*	2.6 (1.7 – 3.9)	< 0.001*
Unknown IN	9 (16.7)	45 (83.3)	1.5 (0.75 – 3.2)	0.245	1.4 (0.68 – 3.0)	0.350
New	352 (11.5)	2705(88.5)			ref	
		HIV Status				
Negative	121(14.2)	731 (85.8)	1.2 (0.72 – 2.1)	0.438	1.2 (0.68 – 2.0)	0.552
Unknown	284 (11.9)	2096 (88.1)	1.0 (0.61 – 1.7)	0.973	1.0 (0.61 – 1.7)	0.926
Positive	18 (11.8)	134 (88.2)			ref	

*statistically significant at $p < 0.05$ ref : reference level. Significant variables on bivariate analysis for potentially unsatisfactory were gender, age, forms of TB, pre-treatment and HIV status were subjected to binary logistic regression with reference indicator as the female gender, children, extra-pulmonary tuberculosis, pre- treatment status of new cases and HIV positive respectively.
