# Supplementary material for: Treatment outcomes of Nigerian patients with tuberculosis: A retrospective 25-year review in a regional medical center
Source: PLoS One. 2020 Oct 29;15(10):e0239225. doi: 10.1371/journal.pone.0239225 (PMC7595370; doi:10.1371/journal.pone.0239225)
Supplement: Attachment — Submitted filename: Response to reviewers 08-22-2020.docx [file pone.0239225.s004.docx]

____________________________________________________________________________________________________________________________________________________________________________________

*Twin Cities Campus*	***Global Pediatrics Program***	*717 Delaware Street SE*

*Department of Pediatrics	Minneapolis, MN	55414*

*Office: 612.626.1980*

*https://med.umn.edu/pediatrics/programs-and-centers/global-pediatrics*

August 20, 2020

**Re- SUBMISSION OF MANUSCRIPT PONE-D-20-11827**

I wish to re-submit the revised manuscript titled “Treatment Outcomes of Nigerian Patients with Tuberculosis: A Retrospective 25-year Review in a Regional Medical Center”

The manuscript has been amended in line with the reviewers’ comments. Kindly find below a list of the reviewers comments and corresponding responses painstakingly made in a tabular format for readability.

Thank you.

Sincerely,

Tina Slusher, MD for Dr. Alao and the team

Tina M. Slusher, M.D., FAAP	Hennepin Healthcare

Professor of Pediatrics	Pediatric Critical Care

Division of Global Pediatrics	701 Park Avenue G7

University of Minnesota	Minneapolis, MN, 55415 USA

Phone 612-840-8883	Fax 612-904-4295

E-mail tslusher@umn.edu

| REVIEWERS COMMENTS | RESPONSE |
| --- | --- |
|  | **General Response**  On behalf of the authors, we appreciate the kind words and the formative assessment from the reviewers. This appears to be one of the most constructive revision the authors have ever experienced. We appreciate the academic editor for the insightful recommendations and thank the journal for the privilege of having excellent reviewers improve the quality of our manuscript. |
| 1. Table 1: is this incidence rate or number of TB cases? The right hand column says "total TB cases" suggesting the latter. | In line with recommendation of the reviewer Table 1 has been changed to a supplementary document now referred to as APPENDIX I. The title has been modified to “Hospital attendance and number of TB cases managed in a regional health centre” |
| 2) Figure 1: unsatisfactory listed twice and numbers not clear. Don't seem to compute. Suggest splitting adult and child outcome data please. | In line with the reviewers comments Figure 1 has been formatted to show the different treatment outcomes in children and adults as suggested. The redundant information has been deleted. |
| 3) Figure 2: remove decimal point and suggest incidence rate is "per 100,000 per year" as opposed to % as this will help the reader and be comparable | The formative suggestion on figure 2 is noted with thanks. The incidence rate has been computed at 100,000 per year as shown in figure 3 in line with the reviewers' comment. |
| 4) Please explain in a bit more detail how adherence was calculated for both intensive and continuation phase (e.g. TB treatment registers, if patients who were LTFU had any measure of adherence measured prior to being LTFU) and if DOTS was maintained throughout this period or whether in continuation phase there was 3x week treatment | Adherence was defined as patients taking ≥80% of the prescribed anti-TB via the DOT intervention or treatment completed uninterrupted.  The study used a daily dosing treatment regimen and calculated adherence for the entire treatment course (no separation was made between the intensive and the continuation phase).  This is admitted as one of limitations of the study. |
| 5) Disease characteristics: I don't think that statistical calculations are necessarily needed for this comparison and don't add much to the raw numbers so would suggest dropping. If you feel strongly that these should stay in then please indicate how the differences in proportion were calculated and present the estimates of error related to these calculations. | The details description on this section have been expunged. The information under this part has been replaced with the sociodemographic and clinical characteristic of the study participants. |
| 6) Proportion of patients with HIV each year would be useful to understand, please can you add this data either in text, integrate into existing table/figure, or new figure/table | A table on TB HIV coinfection has been generated as table 2 Routine screening for infection in presumed tuberculosis cases stated in 2007, thus the proportions of HIV infection from 2007-2017 were shown. |
| 7) Table 3 is unclear. Please can the authors indicate a) what factors were adjusted for in the multivariate analysis? What is "negative" (presumably HIV)? I think tables throughout the manuscript require explanatory legends please. Please can the authors consider whether a separate analysis of the factors associated with treatment adherence and treatment success in a) adults and b) children. This could help to tailor recommendations/advice for different age groups. | The significant variables of sociodemographic characteristics of age (adult or children), gender (male or female) and clinical variables of categories of diseases (pulmonary, extrapulmonary disease, pre-treatment and HIV status on bivariate analysis were subjected to binary logistic regression to adjust for co-variance to determine the predictors of successful treatment outcomes and treatment adherence.  New separate tables (tables 3, 4a- 4c) have been created for each of the four measured treatment outcomes to enhance clarity. An explanatory legends and footnotes have been added to the tables for easy comprehension.  A subgroup analysis for children and adult was omitted because the dummy analysis gave no additional formation as the number of subjects in the paediatric arm was very small. |
| 8) Table 3 also highlights that the authors seem to have omitted how good adherence was calculated. It seems that they used logistic regression so it must be as a dependent binary variable so it would be useful to know what this threshold was. I think the authors should consider whether, given they have the data on adherence, a linear regression model of number of tablets taken / % adherence as the dependent variable might be more fitting. Another consideration would be a time to event (e.g. completing or stopping treatment) would be achievable with the data available. | The thoughtful and expert input of the reviewer is deeply appreciated. Adherence was defined as patients taking ≥80% of the prescribed anti-TB via the DOT intervention or treatment completed uninterrupted.  A time to event/survival analysis is strongly desirable as recommended by the first-rated reviewer. However, the present dataset contained limited information required for this sub-analysis. The authors have agreed to do a robust further study/analysis on adherence from 1991-2020 incorporating the data required for time to event/survival analysis and a linear model of adherence with the number of tablets used by study participants if permitted by the journal who owns the dataset if article is accepted. |
| 9) I appreciate the massive efforts that this follow-up and analysis would have taken. However, given the missing data on treatment outcomes (e.g. a quick calculation of n/N for Table 3 shows there are a significant number of people without a treatment outcome). This could be dealt with in two ways: firstly, the authors could do an analysis of the sociodemographic and clinical characteristics of those with MISSING outcome data; or second, which is more straightforward, is just to mention the missing data in the limitations section and that those with missing data might represent a more vulnerable subgroup of patients. | The apt suggestion by the reviewer is well acknowledged. The missing data is acknowledged as a limitation of this study. |
| 10) All the comments above relating to Table 3 apply equally to Table 4. | New separate tables (tables 3, 4a- 4c) have been created for each of the four measured treatment outcomes to enhance clarity. An explanatory legends and footnotes have been added to the tables for easy comprehension. |
| 11) Referencing needs to be reviewed throughout including formatting of references in main text. | The references have been formatted and the bibliography revised |
| 12) Please can the authors make clear in the methods if they included any interaction terms / likelihood ratio when doing their regression tables. If they did not then suggest that they do this to check for interactions between independent variables. | The recommendation of the reviewer is noted with thanks. In the course of analysis an interaction effects were built from the statistical perspective. Only significant independent variables from the main effects multivariate analyses were interacted. None of the interaction terms were significant thus the results of the main effects were final. We have attached a supplementary table of the interaction terms for reference. |
| L134:”develops smear-positive TB” can be replaced with “has a positive smear” | The suggested comment has been implemented with thanks |
| L137: “Patient dies of any cause” can that be rephrased? Not sure how correctly expressed it is. | The phrase “Patient dies of any cause” has been modified. It now reads  “mortality from any cause” |
| L173: “Testing” can be preferably replaced with “test” | The word testing have been replaced with test |
| L182: “Giving a M:F of 1.3:1”, can be replaced with “giving a male:female ratio of 1.3:1” | The phrase “Giving a M: F of 1.3:1” has been replaced with “Giving a male: female ratio of 1.3:1” |
| L182-183: “Children constituted 332 (9.8%)”, the meaning is not clear. Does it mean that 332 of the study population were children? | The phrase “Children constituted 332 (9.8%)” has been replaced by “332 of the study population were children” for clarity as pointed out by the reviewers. This phrase has been move to the sociodemographic characteristics subsection. |
| L187-188: Numbers should be expressed with words or numbers, so they match the final picture. | This has been under disease characteristic has been re written in line with other reviewers comment and the suggested corrected made into the main text. |
| L219-220: The word “about” should be emitted. | The word “about” has been expunged in line with the formative comment |
| L249-251: Not clear meaning, not clearly written | The confusing sentence has been deleted |
| L254: “this studies higher rate…”. Does it mean “the higher rate of adherence of these studies”? | The phrase “this studies higher rate…”. Has been modified to “the higher rate of adherence of these studies” as suggested |
| L279: “our mean mortality rate” can be rephrased | The phrase “our mean mortality rate” has been modified to The mean mortality rate of 10.0(±3.6) % in our study is |
| L308: “more” can be emitted | The word more has been expunged as suggested |
| Comment on the table with Proportion with TB-Age: Is age expressed in years? Is proportion with TB expressed in %? Please enter the units. | Figure 2 has been labelled with age in years and on the x-axis while the proportion are in percentages. |
| Comment on the flowchart: not sure/unclear the two unsatisfactory results | The figure 1 has been formatted to show the different treatment outcomes among children and adults as suggested. The redundant information has been deleted. |
| General comments:  Please check again the numbers of the references. They are expressed in different ways (mixed). Focus on the discussion. | The references have been checked and formatted in line with the journal's guidelines |
